# Supplementary material for: Multiple transmission routes sustain high prevalence of a virulent parasite in a butterfly host
Source: Proc Biol Sci. 2019 Sep 4;286(1910):20191630. doi: 10.1098/rspb.2019.1630 (PMC6742984; doi:10.1098/rspb.2019.1630)
Supplement: Supplementary material [file rspb20191630supp1.docx]

**Supplementary Material**

*To accompany:* *Multiple transmission routes sustain high prevalence of a virulent parasite in a butterfly host (Majewska,* *Sims, Schneider, Altizer, Hall 2019, Proceedings B; doi:10.1098/rspb.2019.1630)*

***A. General results and detailed statistical analyses of field data on OE infection and spore acquisition***

***OE infection***

The average infection prevalence across the entire sampling period was higher for monarchs captured as larvae (0.57 ± 0.03; *N* =350) relative to monarchs captured as adults (0.38 ± 0.03; *N*= 362; figure 2*a* in main text). The stage of collection (as larva versus adult), and the interaction between stage and month, were significant predictors of infection prevalence (z = 2.00, *p* = 0.05 and z = 2.74, *p* = 0.01, respectively). Males had higher infection probability than females (0.53 ± 0.03, *N* =381 for males; 0.46 ± 0.03, *N* =290 for females; z = 2.32, *p* = 0.02; see table S1 for full model output).

**Table S1.** Full generalized linear model results for predictors of *OE* infection status (0/1) of wild monarchs collected in Savannah, GA garden plots. Significant terms are presented in bold, *p* < 0.05.

| Infection status | Estimate (SE) | z | *p* |
| --- | --- | --- | --- |
| Intercept | **-10.13 (1.59)** | **-6.38** | **1.81 e-10** |
| Month | **1.29 (0.20)** | **6.60** | **4.24 e-11** |
| Sex (males) | **0.43 (0.18)** | **2.33** | **0.02** |
| Stage (larva) | **3.72 (1.86)** | **2.00** | **0.05** |
| Stage x Month | **-0.62 (0.23)** | **-2.74** | **0.01** |

***Spore acquisition by healthy (uninfected) adults***

The probability of spore acquisition and number of spores acquired increased over time (figure 2*b* in main text; z = 3.78 and z= 4.40, *p* <0.001, respectively), but were similar for males and females (z = 0.54 and z = 1.63, respectively, *p* > 0.05; table S2).

**Table S2.** Full generalized linear mixed model results for (a) *OE* spore acquisition status (0/1) and (b) number of spores acquired by healthy adults through adult transfer. Significant terms are presented in bold, *p* < 0.05. Random effect reports the estimated variance and standard deviation for animal id intercept effect.

| (a) Spore acquisition status | Estimate (SE) | z | *p* |
| --- | --- | --- | --- |
| Intercept | **-12.84 (3.65)** | **-3.52** | **4.32 e-4** |
| Month | **1.78 (0.47)** | **3.78** | **1.59 e-4** |
| Sex (males) | 0.44 (0.81) | 0.54 | 0.59 |
| *Random effects* | *Variance* | *Standard Deviation* | |
| Animal ID | 0 | 0 | |
| (b) Number of acquired spores | Estimate (SE) | z | *p* |
| Intercept | **-5.45 (2.19)** | **-2.49** | **0.01** |
| Month | **1.16 (0.26)** | **4.39** | **1.12 e-5** |
| Sex (males) | 0.80 (0.49) | 1.63 | 0.10 |
| *Random effects* | *Variance* | *Standard Deviation* | |
| Animal ID | 0 | 0 | |

***Spore acquisition by milkweed plants***

The proportion of milkweed plants for which at least one monarch emerged as an infected adult increased over time, from zero to approximately 75% (z = 5.84, p < 0.001; table S3).

**Table S3.** Full generalized linear mixed model results for milkweed contamination status (0/1). Significant terms are presented in bold, *p* < 0.05. Random effect reports the estimated variance and standard deviation for plant id intercept effect.

| Milkweed spore status | Estimate (SE) | z | *p* |
| --- | --- | --- | --- |
| Intercept | **-5.93 (0.92)** | **-6.42** | **1.35 e-10** |
| Month | **0.71 (0.12)** | **5.84** | **5.23 e-9** |
| *Random effects* | *Variance* | *Standard Deviation* | |
| Plant ID | 0.216 | 0.4648 | |

***B. Model parameterization***

Below we provide parameters used in model simulations that were estimated using past empirical or field studies (table S4), and details of how some of the parameter values were derived.

**Table S4.** Model parameter definitions, units, values and references from which values were estimated. Derivations of some parameter values are provided below.

| Parameter | Definition | Units | Value | Reference |
| --- | --- | --- | --- | --- |
| *b_S_* | Susceptible host fecundity rate | eggs/adult/day | 4.60 | [1-3] |
| *b_I_* | Infected host fecundity rate | eggs/adult/day | 3.26 | [3] |
| *τ_e_* | Time in egg stage | days | 3 | [4] |
| *g* | Larval development rate | 1/day | 1/9 | [4] |
| *µ_0_* | Density-independent *per capita* larval mortality rate | 1/day | 0.31 | *See below for derivation* |
| *µ_d_* | Density-dependent *per capita* larval mortality rate | 1/day | 1370 | *See below for derivation* |
| *τ_p_* | Time in pupa stage | days | 7 | [4] |
| *ρ* | Probability of pupa surviving to adult |  | 0.76 | [5] |
| *θ* | Probability of infected adult eclosing and mating |  | 0.72 | [6] |
| *µ_S_* | Mortality rate of uninfected adult | 1/day | 1/24 | [2] |
| *µ_I_* | Mortality rate of infected adult | 1/day | 1/20 | [2] |
| *p_v_* | Probability of vertical transmission |  | 0.90 | [7] |
| *p_h_* | Probability of contaminated adult infecting their offspring |  | 0.614  Range:  0.45 - 0.9 | [7] |
| δ | Daily adult mating probability | 1/day | 1/2 | [8] |
| *µ_C_* | Rate of spore loss for contaminated adults | 1/day | 1/ 14  Range:  1/1 - 1/15 | [7] |
| *r* | Milkweed growth rate | leaves/day | 0.032 | *this study* |
| *K* | End of season number of milkweed leaves (assuming 200 leaves per plant) | leaves | 40000 | *this study* |
| *c* | Larval consumption rate of milkweed | leaves/day | 3.889 | [9] |
| *λ* | Milkweed leaf visitation rate by infected adults (number of visits resulting in deposition of an infectious spore dose) | leaves/day | 50  Range:  1 - 200 | *this study* |
| *µ_w_* | Spore decay rate on milkweed | 1/day | 1/80  Range:  1/1 - 1/80 | [9] |
| *S_0_* | Initial uninfected adult monarch population | adults | 18 | *this study* |
| *I_0_* | Initial infected monarch population | adults | 2 | *this study* |
| *C_0_* | Initial number of uninfected adults that acquire spores through adult transfer | adults | 0 | *this study* |
| *M_0_* | Initial number of spore-free milkweed leaves at site | leaves | 7000 | *this study* |
| *M_C0_* | Initial number of spore-contaminated milkweed leaves | leaves | 0 | *this study* |
| *T* | Model run time (from site recolonization by monarchs until prior to fall migrants) | days | 150 | *this study* |

***Monarch fecundity***

The susceptible host fecundity rate per adult monarch, *b_S_*, was calculated as the product of proportion of females in population, lifetime egg production/adult lifespan, and the probability that an egg survives until a first instar larva. We assumed a 50:50 sex ratio, calculated lifetime egg production (442 eggs) as the average reported by three studies [1-3], a healthy adult lifespan of 24 days [2], and we assumed the probability of egg survival is 0.5. The infected host fecundity rate *b_I_* accounted for a 41% reduction in total egg production [6], and an average infected adult lifespan of 20 days [2]. Adult lifespan estimates are consistent with the range of the lifespans we observed in the field in this study (12-30 days). We therefore estimated *b_S_* = 4.60 and *b_I_* = 3.26 eggs per adult per day.

***Development of immature stages***

To calculate time spent in egg, larval and pupal stages, we use the degree-day model as described by [4], using 28°C as a typical daytime temperature over 5 month observation period at our field site in Savannah, GA, USA. We incorporate the time spent in the egg and pupal stages as developmental delays, where all individuals spend exactly $\tau_{e}$ = 3 and $\tau_{p}$ = 7 days in these stages respectively. The development time for larvae that survive from egg to pupation is 9 days; we therefore assume a larval development rate of *g* = 1/9 days.

***Larval mortality***

We followed the method described in [9] to estimate density-dependent and density-independent per capita larval mortalities. The density-independent mortality rate *µ_0_* is related to the probability of surviving the 9-day period from egg to pupation, *s*, such that

$$s=e^{-\mu_{0}*9}$$

Solving the equation for *µ_0_* density-independent mortality yields:

$$\mu_{0}=- \frac{\ln s}{9}$$

We used estimates of egg to 5^th^ instar survival based on our field observations, equal to 0.06, which is similar to survival estimates reported previously [5]. With *s* = 0.06, density-independent larval mortality rate $\mu_{0}$=0. 31.

Density-dependent mortality *µ_d_,* is derived by using disease-free host dynamics as represented by a system of differential equations for larval (*N_L_*) and adult abundance (*N_A_*)*,* and then solving the equations for *µ_d_* at equilibrium:

$$\frac{dN_{L}}{dt}= b_{S}N_{A}-\left. {\left( \mu_{0}+\mu_{d}\frac{N_{L}}{M} \right)N}_{L}-g \right.N_{L}$$

$$\frac{dN_{A}}{dt}= g\rho N_{L}-{\mu_{S}N}_{A}$$

Where *ρ* represent survival probability from pupation to eclosion. We incorporate loss of pupa by multiplying *g* by *ρ* =0.76 to reflect probably of pupa surviving to eclosion [5].

When *dN_L_/dt and dN_A_/dt* are equal to 0:

$$\mu_{d}=\left( \frac{M}{N_{L}} \right) \left. \left( \frac{b_{S} g \rho}{\mu_{S}}-\mu_{0}-g \right) \right.$$

We use our late-season field estimates of larval density per plant (1.3) and number of milkweed leaves per plant (200) to approximate the equilibrium value of $\frac{M}{N_{L}}$ is 153.8 in the expression above. Substituting the remaining derived parameter values (see table S4 above) in the expression above yields the density-dependent larval mortality rate *µ_d=_*1370*.*

***Milkweed consumption***

We assume monarch larvae consume milkweed leaves at a constant per capita rate *c*, as reported by [9], which reflected the total number of *Asclepias incarnata* milkweed leaves (a related and morphologically similar milkweed species) consumed per monarch larva. Therefore, we calculated *c* equal to 35 leaves divided by the 9-day time period in larval stage, *c* = 3.89 leaves per day.

***Costs of infection on eclosion and mate-finding***

Experimental work shows that relative to an uninfected monarch, the probability of an infected adult eclosing is 0.9, and the probability of a heavily infected monarch mating successfully is 0.8 [3]. We therefore use the product of the two probabilities to yield *θ* = 0.72 as the probability of heavily infected monarch mating and eclosing successfully.

***Environmental transmission and adult transfer***

We model environmental transmission following [9]. Briefly, infected adults shed spores onto milkweed leaves when adults visit milkweed plants to nectar or oviposit at a constant per capita rate *λ*. Based on field observation, a monarch contacts milkweed approximately 30 times per hr. Assuming monarchs are active for 7 hrs a day and that each contact results in shedding of infectious dose of spores to 1 leaf, we estimate this visitation rate to equal about 200 milkweed leaves per day. Since there is uncertainty about variation in plant visitation rates among monarchs, and the probability that one visit results in deposition of an infectious spore dose, we perform extensive sensitivity analyses varying this parameter (*λ* = 1 – 200; see section C below).

To model adult spore transfer, we assume that the daily probability of adults mating, *δ =* ½ (based on [8]), and that the probability a susceptible monarch mates with an infected monarch equals the proportion of infected adults in the population$\left( \frac{I_{A}}{N_{A}} \right)$. Experimental work shows that contaminated adults *C_A_* lose acquired spores over time [7]. Based on field observations we assume some spores are retained for 14 days, yielding *µ_C_* the rate of spore loss equal to 1/14. Because previous work shows that most spores can be lost as quickly as 5 days [22], we also we perform sensitivity analyses and vary spore retention, or 1/ *µ_C_* (1/1 – 1/15; see section C below).

***Initial conditions***

We set initial conditions in the model to reflect our field scenario of a tropical milkweed patch that has just been recolonized in the spring following a winter die-back of monarchs and milkweed. Therefore, we assume that initially there are no larvae (*S_L_* = *I_L_* = 0) and no spores on milkweed (*M_C0_=*0). We let initial number of leaves, *M_0_ = 7*000, based on our field observations. We assume 20 adults colonize a patch of tropical milkweed start of the season (time *T =* 0) with only 10% of infected individuals (based on our field observation; figure 2*a* in main text). Therefore, initial infection prevalence is equal to 10%, initial numbers of susceptible adults *S_A_* = 18, and initial numbers of infected adults *I_A_ =* 2.

***Virulence***

The lifetime reproductive success of susceptible (uninfected) adult can be defined as the fecundity rate of a susceptible adult over its adult lifespan:

LRS_S_ = $\frac{b_{S}}{\mu_{S}}$

Similarly, lifetime reproductive success of an infected adult can be defined as the fecundity rate of infected adult over its lifespan, incorporating infection-induced reductions in lifespan, egg production, and the reduced probability of eclosion and mating ($\theta$):

LRS_I_ = $\frac{b_{I}\theta}{\mu_{I}}$

We define a composite measure of virulence as the proportionate reduction in lifetime reproductive success of infected adults relative to susceptible adults:

*virulence* = $1-\frac{{LRS}_{I}}{{LRS}_{S}}=1-\frac{b_{I}}{b_{S}}$ $\frac{\mu_{S}}{\mu_{I}}$ $\theta$

***C. Sensitivity analyses***

We explored the sensitivity of model outcomes to variation in the parameters that dictate environmental transmission, adult transfer, and virulence (figures S1-S2). Increasing parameters relating to the rates of transmission (i.e., milkweed visitation rate, λ, and the probability that contaminated adults infect their offspring, *p_h_*,) or duration of contamination (i.e., the duration of spore persistence on milkweed, 1/ *µ_w_*, or spore-contaminated adults, 1/ *µ_C_*) had similar qualitative effects of increasing late-season prevalence and decreasing host population size. However, the magnitude of these effects was stronger for changes in transmission rates than changes in spore duration. Increasing adult transfer had stronger effects on prevalence than increasing environmental transmission, although increasing environmental transmission had larger negative effects on host abundance (figures S1*a-d*). The most severe reductions in adult abundance, and increases in late-season prevalence occurred when both transmission rates were high (figures S1*e-f*). For both transmission routes, the largest impact of infection on host populations occurred when virulence was higher than our baseline scenario (i.e., causing an 80% reduction in relative fitness of infected hosts), whereas prevalence was maximized at lower virulence (figures S2). Finally, we performed global sensitivity analyses utilizing Latin Hypercube Sampling (figure S3).

a. b.


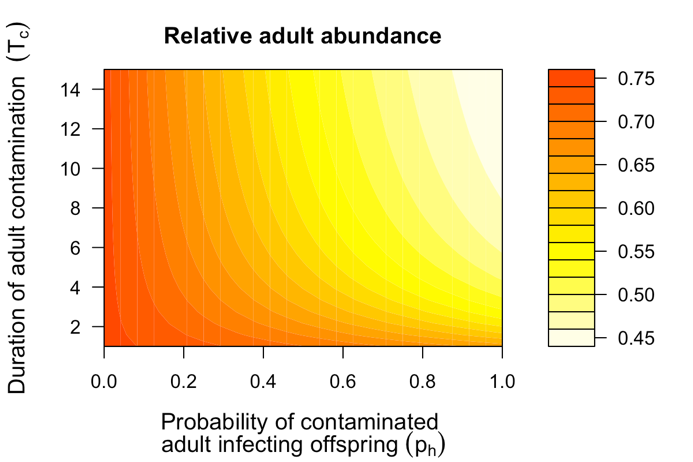

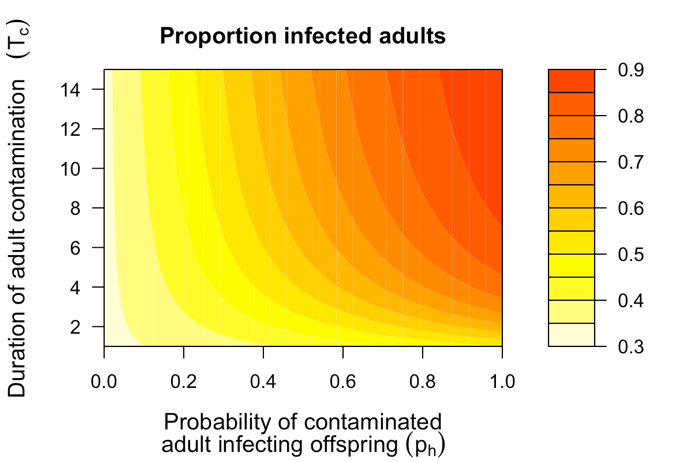


c. d.


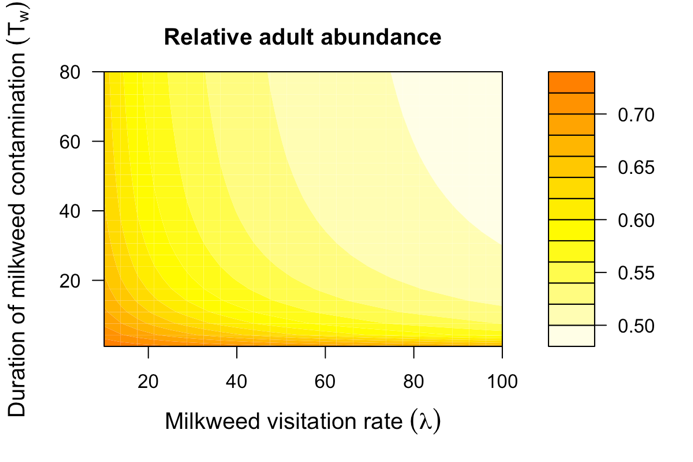

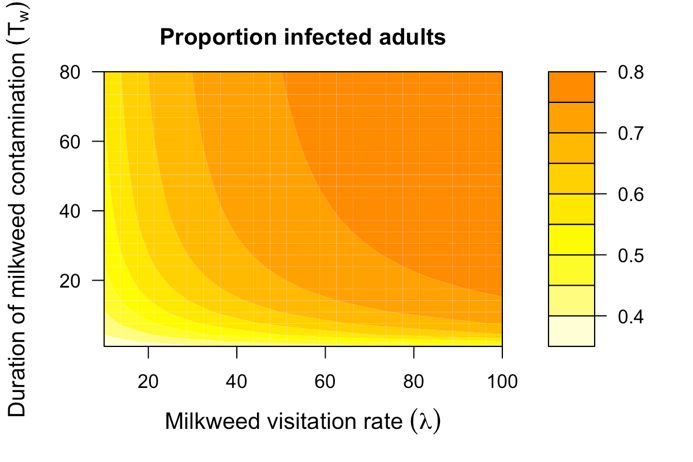


e. f.

**
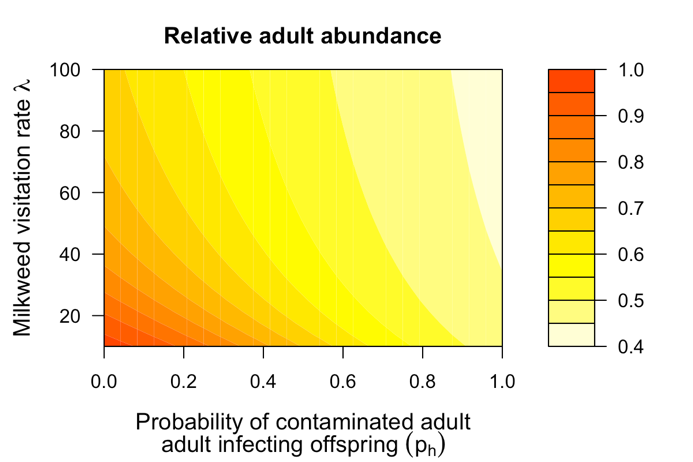

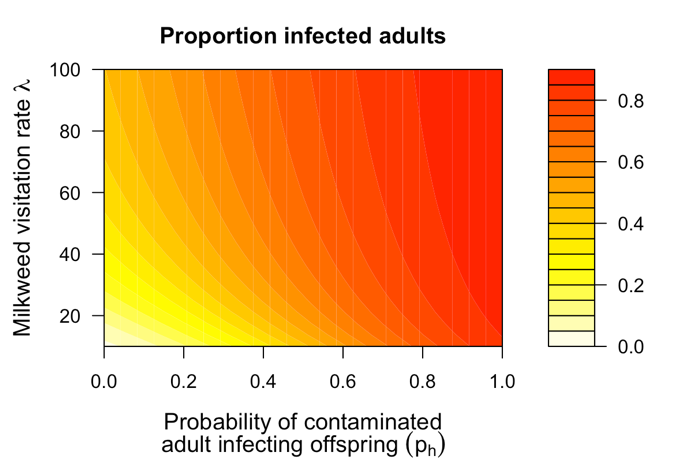
**

**Figure S1.** Heat contours for predicted adult abundance (relative to a parasite-free population size) and proportion of infected adults at the end of season (150 days) for (a-b) range of days contaminated adults retain spores (*T_C_* = 1/*μ_C_*) and the probability that contaminated adult infects own offspring (*p_h_*); (c-d) range of days contaminated milkweed retain spores (*T_W_*= 1/*μ_w_*) and milkweed visitation rate (λ); and (e-f) milkweed visitation rate (λ) and probability that contaminated adult infects own offspring (*p_h_*).

a. b.


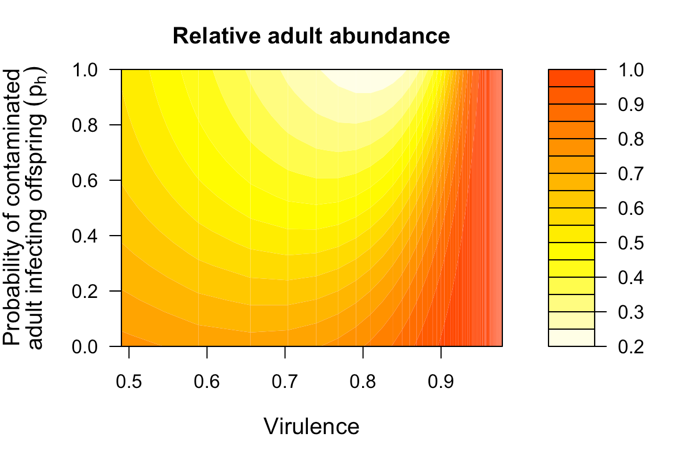

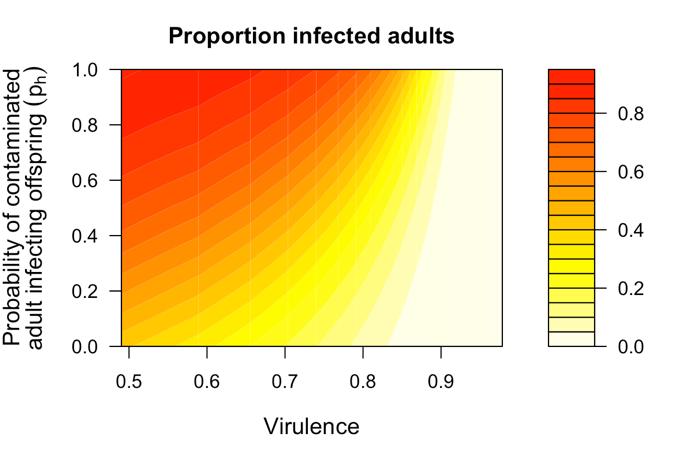


c. d.


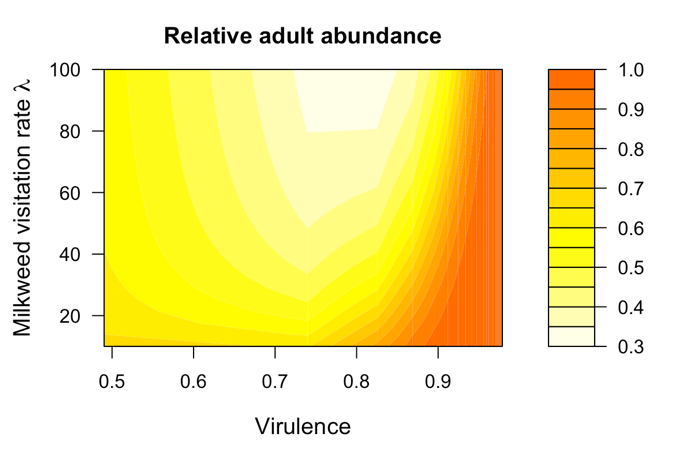

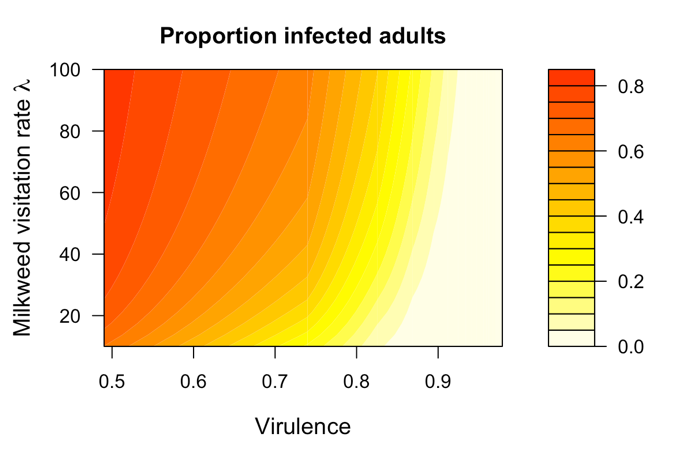


**Figure S2.** Results of the model shown in heat contours for predicted relative adult abundance (relative to a disease-free population size) and proportion of adults infected with *OE* at the end of 150-day season as a function of (a-b) the probability that contaminated adult infects own offspring (*p_h_*) and virulence; (c-d) milkweed visitation rate λ and virulence.

***Latin Hypercube Sampling***

We also employed Latin Hypercube Sampling for global sensitivity analyses. Specifically, we used the R package *lhs* [10] to generate 500 samples from a Latin hypercube design in which parameters were distributed uniformly. We varied parameters with greatest uncertainty and those we suspected would impact model outcomes (table S5). To derive partial rank correlation coefficients between parameters and the equilibrium (end of 150-day season) proportion of infected adults we used R package *sensitivity* [11].

**Table S5.** Ranges for parameters we varied in the Latin hypercube sampling procedure.

| **Parameter** | **Definition** | **Range** |
| --- | --- | --- |
| b_S_ | susceptible host fecundity rate | 2.85 – 7.50 |
| µ_0_ | density-independent larval mortality rate | 0.120 – 0.4 |
| L | larval density per plant which yields density-dependent larval mortality rate *µ_d_* | 0.25 - 3 |
| p_h_ | probability of contaminated adult infecting their offspring | 0 - 1 |
| T_C_ | number of days transferred spores remain on contaminated adults, or *1/ µ_C_* | 1 - 15 |
| λ | visitation rate by infected adults which results in infectious spore dose and new infection | 1 - 200 |
| T_W_ | number of days a spore-contaminated milkweed leaf remains infectious (inverse of spore decay rate, *1/ µ_w_*) | 1 - 100 |

a.

**
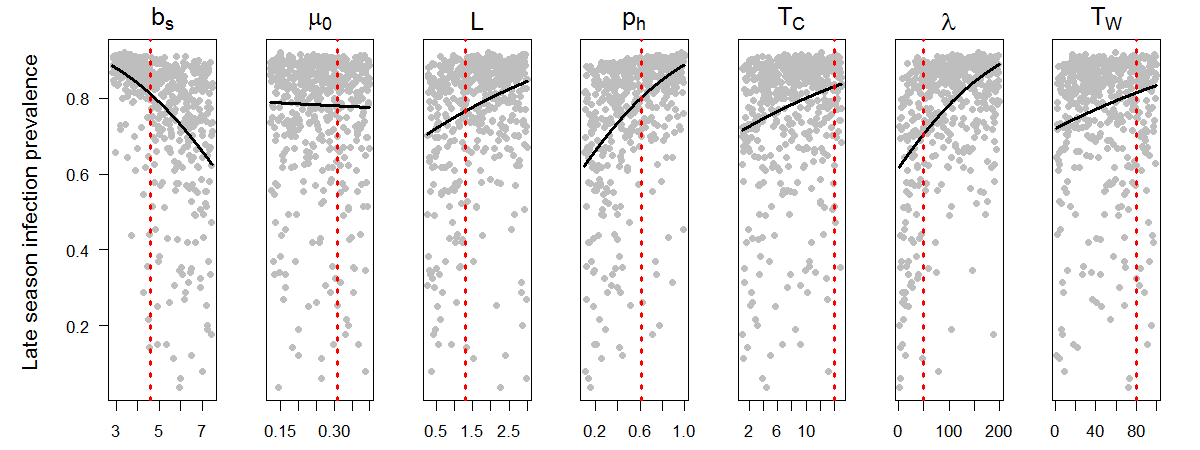
**

b.

**
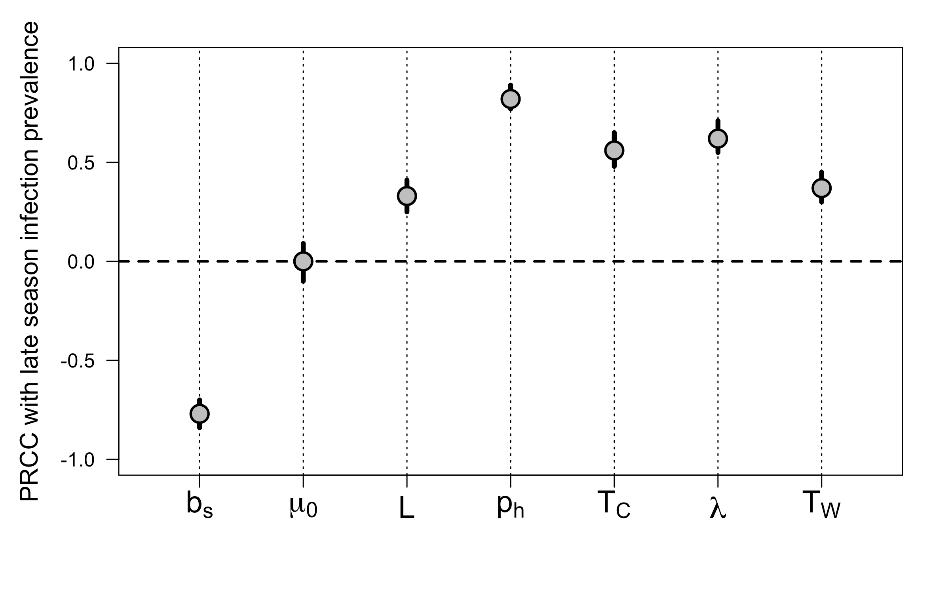
**

**Figure S3.** (a) Predicted equilibrium (end of season) proportion of adults infected as larvae with varying parameter values (table S5 above) using Latin Hypercube Sampling to sample the parameter space. Dashed red lines show default parameter values used for the main text results (see table S4 for exact values). (b) Partial Rank Correlation Coefficient (PRCC) sensitivity analysis. PRCC values indicate the strength and direction of association between model parameters and the end of season proportion of adults infected as larvae.

**References**

[1] Oberhauser, K.S. 1997 Fecundity, lifespan and egg mass in butterflies: effects of male‐derived nutrients and female size. *Funct. Ecol.* **11**, 166-175.

[2] Altizer, S. & Oberhauser, K.S. 1999 Effects of the protozoan parasite *Ophryocystis elektroscirrha* on the fitness of monarch butterflies (*Danaus plexippus*). *J. Invertebr. Pathol.* **74**, 76-88.

[3] De Roode, J.C., Chi, J., Rarick, R.M. & Altizer, S. 2009 Strength in numbers: High parasite burdens increase transmission of a protozoan parasite of monarch butterflies (*Danaus plexippus*). *Oecologia* **161**, 67-75.

[4] Zalucki, M.P. 1982 Temperature and rate of development in *Danaus plexippus* L. and *D. chrysippus* L. (Lepidoptera: Nymphalidae). *Aust. J. Entomol.* **21**, 241-246.

[5] Nail, K.R., Stenoien, C. & Oberhauser, K. 2015 Immature monarch survival: Effects of site characteristics, density, and time. *Ann. Entomol. Soc. Am.* **108**, 680-690.

[6] De Roode, J.C., Yates, A.J. & Altizer, S. 2008 Virulence-transmission trade-offs and population divergence in virulence in a naturally occurring butterfly parasite. *Proc. Natl. Acad. Sci.* **105**, 7489-7494.

[7] Altizer, S., Oberhauser, K.S. & Geurts, K.A. 2004 Transmission of the protozoan parasite, *Ophryocystis elektroscirrha*, in monarch butterfly populations: implications for prevalence and population-level impacts. In *The monarch butterfly: Biology and conservation* (eds. K.S. Oberhauser & M. Solensky), pp. 203-218. Ithaca, NY, Cornell University Press.

[8] Oberhauser, K.S. 1989 Effects of spermatophores on male and female monarch butterfly reproductive success. *Behav. Ecol. Sociobiol.* **25**, 237-246.

[9] Satterfield, D.A., Altizer, S., Williams, M.-K. & Hall, R.J. 2017 Environmental persistence influences infection dynamics for a butterfly pathogen. *PLoS One*, e0169982.

[10] Carnell, R. 2017 R package lhs: Latin Hypercube Samples, v. 0.16

[11] Iooss, B., Janon, A. & Pujol, G. 2016 R package sensitivity: Global Sensitivity Analysis of Model Outputs, v. 1.15.2
